# Does Digital Make a Difference? Willingness‐to‐Pay for Digital Versus Offline Weight Loss in Germany

**DOI:** 10.1002/osp4.70151

**Published:** 2026-05-21

**Authors:** Kevin Helms, Stefan K. Lhachimi, Wolf Rogowski, Oliver Lange

**Affiliations:** ^1^ Department of Health Care Management Institute for Public Health and Nursing Sciences Health Sciences University of Bremen Bremen Germany; ^2^ Leibniz ScienceCampus Digital Public Health Bremen Germany; ^3^ Department of Nursing Management University of Applied Sciences Neubrandenburg Neubrandenburg Germany

**Keywords:** contingent valuation, digital public health, economic evaluation, weight loss, willingness‐to‐pay

## Abstract

**Background:**

Germany introduced both digital health applications (DiGA) and an “offline” structured disease management program (DMP) for obesity within statutory health insurance. Given limited resources and ongoing reimbursement decisions, evidence on how individuals with obesity assessed the value of intervention delivery in terms of their willingness to pay (WTP) remained limited.

**Objective:**

This study assessed the WTP of individuals with obesity (BMI ≥ 30 kg/m^2^) for a DiGA and a structured “offline” DMP and examined whether digital interventions better aligned with their preferences than offline interventions.

**Methods:**

An online contingent valuation survey was conducted among 424 participants with a BMI ≥ 30 kg/m^2^ in Germany. Monthly WTP under a hypothetical non‐coverage scenario was analyzed in relation to participants' demographic and socioeconomic characteristics using a two‐part regression model.

**Results:**

The mean WTP for the DiGA was €16.15 per month (95% CI: €14.22–€18.09) and was significantly positively associated with older age (€0.24 higher per year, *p* = 0.02) and higher digital affinity (€6.91 higher per unit, *p* = 0.01). The mean WTP for the DMP was €18.70 per month (95% CI: €16.52–€20.90) and was significantly positively associated with female sex (€6.20 higher, *p* = 0.02) and higher digital infrastructure (€2.63 higher per unit, *p* = 0.04).

**Conclusion:**

Respondents showed a positive WTP for weight loss interventions, yet at an amount far below DiGA manufacturer prices of approximately €70 per month. The small difference between digital and offline formats suggested that the perceived effectiveness of the intervention had a greater influence on WTP than the format.

## 1 Introduction

In Germany, the prevalence of obesity (defined as BMI ≥ 30 kg/m^2^) among adults reached 19% by 2022, affecting nearly 13 million people [1]. The direct social costs of obesity on the German health sector were estimated at over €63 billion annually as early as 2015 [2]. However, cost estimation is fraught with great uncertainty, and the literature on obesity cost estimation reveals a heterogeneous picture [3]. The rise in obesity and chronic diseases underscores the need for changes in health service provision [4].

The digitalization of healthcare is considered a forward‐looking approach, as it may offer numerous opportunities for prevention, patient‐centered care, and efficiency gains [5]. Though still emerging, various digital services already support disease prevention [6, 7], self‐management [8, 9], rehabilitation [10, 11], teleconsultations [12, 13] and remote monitoring [14, 15]. The digital transformation of the healthcare system is gaining increasing importance at the political level in Germany [16]. With the Digital Healthcare Act, Germany became the first country worldwide to allow patients to obtain certified digital health applications (DiGA) by prescription, with their costs covered by statutory health insurance [17]. Unlike typical health apps, DiGAs undergo strict safety, functionality, and outcome assessments by the Federal Institute for Drugs and Medical Devices (BfArM). For obesity treatment, approved DiGAs such as Zanadio and Oviva use multimodal interventions that combine dietary guidance and activity tracking to support long‐term behavioral change.

Besides these digital options, there are also offline interventions, such as the disease management programs (DMP). The DMP Obesity was introduced in Germany in 2024, following the 2023 decision of the Federal Joint Committee (G‐BA). For the first time, it combines different obesity treatment approaches in a comprehensive nationwide care program within the statutory health insurance system [18]. However, the DMP for obesity has not been fully implemented to date. Similar to DiGA, this program delivers structured, evidence‐based care to achieve sustainable weight reduction and stabilization through behavioral change. However, unlike DiGAs, the DMP promotes coordination between general practitioners and specialists, includes structured training and individualized treatment goals, and involves continuous documentation by physician practices.

The number of digital health interventions is rising, and given limited resources in healthcare, there is a need to decide which new (digital) intervention should be reimbursed [19]. Cost‐benefit, as one type of economic evaluation, might be a method to assess whether new interventions provide good value for money. Therefore, the willingness to pay (WTP) could be elicited. If WTP exceeds the intervention's costs, it is assumed to provide a potential Pareto improvement, and thus, good value for money [20]. While WTP for health services has been widely studied [21], few studies have examined WTP for weight loss interventions. A systematic review from 2024 identified nine studies on WTP for overweight and obesity interventions in adults, reporting WTP values ranging from $10 to $45 per month [22]. To the authors' knowledge, no study has assessed WTP for a digital weight loss intervention. The objective of this study was to compare WTP for digital and offline weight‐loss interventions among individuals with a BMI of 30 kg/m^2^ or higher and to examine factors associated with WTP.

## 2 Methods

An online survey was conducted from a personal ex ante perspective, capturing patients' views by asking individuals with obesity how much they were willing to pay for a DiGA or a structured DMP to treat obesity. Besides eliciting the WTP, the effects of various factors, including age, sex (biological sex; recorded at panel enrollment and limited to female and male), net equivalent income, health status (EQ‐5D‐5L), digital affinity, quality of digital infrastructure, and medical care structure, on the stated WTP were investigated.

### 2.1 Sample and Data Collection

A survey was developed using a contingent valuation approach. The survey was conducted by the independent research institute forsa via the forsa.omninet panel, which is representative of German internet users aged ≥ 18 years and recruited offline via random telephone sampling. Participants with a BMI of 30 kg/m^2^ or higher living in Germany were invited in sequential batches and were incentivized with bonus points redeemable as vouchers or for donation. The final survey was conducted between March 7 and March 15, 2024, following a pretest with 56 participants.

### 2.2 Questionnaire

The questionnaire included contextual information, WTP questions, and questions about the expected explanatory variables of WTP (see Supporting Information S1). In the contextual information, textual and visual materials were used to provide information on (1) clinical aspects of obesity; (2) the function and aims of DiGAs in general and DiGAs for the treatment of obesity; and (3) the function and aims of DMPs.

For the WTP questions, payment scales (PS) were used as the elicitation format. To minimize value cue biases, participants were randomly assigned to one of two PSs, both ranging from €0 to “more than €100” but with different distributions of predefined values. The PS design was informed by typical digital health app prices. Popular apps like WeightWatchers, Noom, and MyFitnessPal cost approximately €10 per month (annualized), while obesity‐focused DiGAs like Oviva and Zanadio cost approximately €70 per month for self‐paying users. PS 1 used €10 as the central value, while PS 2 used €40 (the mean of app and DiGA prices). The €100 upper limit reflects the upper range of prices for weight‐loss DiGAs, allowing for some upward flexibility.

The WTP questions referred to a hypothetical non‐reimbursement scenario in which neither the DiGA nor the DMP was covered by health insurance. Each participant was asked the same two questions: which monthly amount they would definitely be willing to pay for the obesity intervention, and which monthly amount they would definitely not be willing to pay. If participants selected the highest value, their WTP was elicited using an open‐ended question. After receiving the answers, the determined WTP was reported and participants were provided with the opportunity to correct the values. Respondents were also allowed to provide explanations for their chosen WTP in a free‐text box. Individual WTP was calculated as the average of the two stated amounts. The mean WTP was then calculated by dividing the sum of all individual WTP values by the number of participants.

To determine which factors influence WTP, information on several variables was collected, including contextual questions about digital infrastructure, digital affinity, health and medical care structure, as well as demographic and socioeconomic characteristics. Health‐related quality of life was surveyed using the EQ‐5D‐5L [23]. The hypothesized impact of each variable on WTP is presented in Table 1.

**TABLE 1:** Hypothesized influences of individual and context characteristics on WTP.

Explanatory variables	Hypothesis on expected WTP
Age	Younger respondents are more familiar with digital interventions and have a higher WTP for DiGA than for DMP; in contrast, older respondents have a higher WTP for DMP than for DiGA.
Sex	WTP may differ by sex due to sex‐related differences in attitudes and experiences related to weight and health.
General health status (EQ‐5D‐5)	Respondents with better general health express a lower WTP for weight loss interventions, as they are likely to derive less benefit from the interventions.
Digital affinity	Respondents with a higher digital affinity express a higher WTP for DiGAs than respondents with a low digital affinity.
Digital infrastructure	Respondents with a good internet connection express a higher WTP for DiGAs as they can use the app without any problems.
Medical care structure	Respondents with an inadequate medical care structure express a higher WTP for DiGAs, as apps can compensate for the lack of medical care
Net equivalent income	Higher income is positively correlated with higher WTP, as increased ability to pay influences WTP.
Education	A higher level of education leads to a higher WTP, as a better understanding of the disease and its risks increases the appreciation of the interventions.

### 2.3 Data Analyses

To investigate WTP for DiGA and a DMP, a two‐part regression model was applied. The first part estimated the probability of a positive WTP (probit model). The second part included only those observations with a positive WTP in a general linear model (GLM) with a gamma distribution, which accounted for the highly skewed WTP distribution. The modeling choice was validated using a modified Park test. Marginal effects on expected WTP were derived using the delta method. In addition, differences in WTP between the DiGA and the DMP were examined using a Wilcoxon matched‐pairs signed‐rank test, as the WTP variables violated normality assumptions. All analyses were conducted in STATA 17 [24].

To ensure robustness, the reasons participants provided for their WTP were analyzed independently by two reviewers. Based on this analysis, categories of potential bias were developed. These included: (1) claims that healthcare should be free in general; (2) claims that costs should be covered by third parties (e.g., health insurance); (3) statements emphasizing altruistic motives (e.g., that costs should be low so that everyone can afford access); and (4) justifications based on misconceptions about the intervention (e.g., assuming that both interventions were digital). Both researchers assigned each justification to one of these categories, and any discrepancies were resolved through discussion until consensus was reached. Justifications assigned to any of these categories were marked as potentially biased and considered for exclusion.

### 2.4 Ethics Approval

This study was approved by the ethics commission of the University of Bremen (reference number 2015‐35).

## 3 Results

### 3.1 Descriptive Analysis

A total of 424 respondents with a BMI of 30 kg/m^2^ or higher completed the survey. Based on the assessment of potentially bias‐indicative justifications for their WTP statements, 53 respondents were excluded from the analysis, resulting in a final analytical sample of 371 participants.

The mean age of the 371 included participants was 52 years (SD 10.95). Of these, 38.8% identified as female (*n* = 144) and 61.2% as male (*n* = 227). The mean digital affinity index score (range −1 to 1; calculation details are provided in Supporting Information S2) was 0.49 (SD 0.43). The mean health status, measured by the German EQ‐5D‐5L tariff (range 0–1), was 0.87 (SD 0.17). Respondents rated the accessibility of healthcare services on a 1 (very positive) to 5 (very negative) scale; the mean rating was neutral, 3.18 (SD 1.04). The quality of digital infrastructure was assessed on a scale of 1 (very poor) to 5 (very good), with a mean rating of 3.57 (SD 0.96), indicating a neutral to good quality. On average, participants had nearly 12 years of education (SD 2.68), and 44.6% reported an equivalized monthly net income of ≥ €2400 (*n* = 149). Table 2 presents an overview of the characteristics of the included and excluded respondents. For further details on the operationalization of the descriptive variables, see Supporting Information S2.

**TABLE 2:** Characteristics of the included and excluded respondents.

	Included sample	Excluded due to potential bias	*p*‐values	Total
Respondents	371	53		424
Payment scales				
Payment scale 1	194 (52.3%)	25 (47.2%)		219 (51.7%)
Payment scale 2	177 (47.7%)	28 (52.8%)		205 (48.3%)
Digital affinity (index)	0.49 (0.43)	0.54 (0.45)	0.41	0.49 (0.43)
Medical infrastructure	3.18 (1.04)	2.83 (1.05)	0.02	3.14 (1.05)
Digital infrastructure	3.57 (0.96)	3.38 (1.06)	0.17	3.55 (0.97)
Age	52.39 (10.95)	50.43 (9.27)	0.22	52.14 (10.77)
Sex			0.02	
Male	227 (61.2%)	41 (77.4%)		268 (63.2%)
Female	144 (38.8%)	12 (22.6%)		156 (36.8%)
Health (EQ‐5D‐5L)	0.87 (0.17)	0.86 (0.17)	0.84	0.87 (0.17)
Education in years	11.88 (2.68)	12.69 (2.80)	0.04	11.98 (2.70)
Equalized net income ≥ €2400	149 (44.6%)	28 (54.9%)	0.17	177 (46.0%)

*Note:* Values are presented as mean (SD) for continuous variables and *n* (%) for categorical variables. *p*‐values refer to comparisons between included and excluded respondents.

The 53 respondents who were excluded due to potentially biased justifications differed from the included sample in a few characteristics. Specifically, they had a higher proportion of males (77.4%) compared with 61.2% (*p* = 0.02), reported slightly higher average education of 12.69 years (SD 2.80) compared with 11.88 years (SD 2.68; *p* = 0.04), and indicated lower medical infrastructure scores of 2.83 (SD 1.05) compared with 3.18 (SD 1.04; *p* = 0.02). No significant differences were observed between the two groups in terms of digital affinity, digital infrastructure, age, health (EQ‐5D‐5L), income, or the distribution of PS.

### 3.2 WTP Outcomes

The WTP for the DiGA was assessed among 344 participants, with an average WTP of €16.15 (SD 18.21). PS 1 had a mean WTP of €16.23 (SD 19.59; 182 observations), and PS 2 had a mean WTP of €16.06 (SD 16.58; 162 observations). For the DMP, 333 participants were considered, with a mean WTP of €18.70 (SD 20.34). On PS 1, the WTP was €17.95 (SD 21.42; 177 observations), and on PS 2 it was €19.57 (SD 19.07; 156 observations).

The mean WTP for the DMP was €2.55 higher than the mean WTP for the DiGA, corresponding to a 15.8% increase. At the same time, 176 respondents (47.4%) reported equal WTP for both interventions. A Wilcoxon matched‐pairs signed‐rank test was conducted due to the non‐normal distribution of the WTP variables; the test showed that the difference in WTP was statistically significant (*p* < 0.01). An overview of the mean WTP values (SD) for the DiGA and the DMP, by PS, is presented in Table 3.

**TABLE 3:** Mean WTP for DiGA and DMP.

	WTP for DiGA			WTP for DMP		
	Obs.	Mean (SD)	(95% CI)	Obs.	Mean (SD)	(95% CI)
Scale 1	182	€16.23 (19.59)	(13.38; 19.09)	177	€17.95 (21.42)	(14.78; 21.12)
Scale 2	162	€16.06 (16.58)	(13.50; 18.63)	156	€19.57 (19.07)	(16.56; 22.57)
Total	344	€16.15 (18.21)	(14.22; 18.09)	333	€18.70 (20.34)	(16.52; 20.90)

Abbreviations: CI = Confidence Interval, Obs. = Observations, SD = Standard Deviation.

Regression analyses were conducted to identify determinants of WTP for the DiGA and the analog DMP weight‐loss interventions. An overview of the regression results is provided in Table 4. For the DiGA, the probit model revealed a significant negative association between age and the likelihood of being willing to pay (*p* = 0.03), while no other variables showed significant effects. In contrast, the GLM model indicated that both higher age (*p* < 0.01) and stronger digital affinity (*p* = 0.01) were significantly associated with higher WTP amounts. For the DMP, the probit model similarly showed a negative association between age and the likelihood of being willing to pay (*p* = 0.03). In the GLM model, female sex (*p* = 0.03) and higher digital infrastructure (*p* = 0.04) were both significantly associated with higher WTP amounts.

**TABLE 4:** Determinants of WTP for DiGA and DMP.

	WTP for DiGA						WTP for DMP					
	Probit‐model (*n* = 295)			GLM (*n* = 283)			Probit‐model (*n* = 287)			GLM (*n* = 274)		
	Coeff.	(95% CI)	*p*‐values	Coeff.	(95% CI)	*p*‐values	Coeff.	(95% CI)	*p*‐values	Coeff.	(95% CI)	*p*‐values
Digital affinity (index)	0.15	(−0.58; 0.88)	0.69	0.40	(0.09; 0.71)	0.01	0.12	(−0.60; 0.83)	0.75	0.24	(−0.08; 0.55)	0.14
Medical infrastructure	0.20	(−0.08; 0.48)	0.16	−0.00	(−0.12; 0.12)	0.99	0.14	(−0.12; 0.40)	0.30	0.00	(−0.12; 0.12)	0.95
Digital infrastructure	0.10	(−0.21; 0.41)	0.54	0.07	(−0.06; 0.19)	0.28	0.08	(−0.22; 0.37)	0.61	0.13	(0.00; 0.25)	0.04
Age	−0.04	(−0.07; 0.00)	0.03	0.02	(0.01; 0.03)	0.00	−0.04	(−0.07; −0.00)	0.03	0.00	(−0.01; 0.01)	0.67
Sex (female)	0.41	(−0.27; 1.08)	0.24	0.22	(−0.02; 0.47)	0.07	0.47	(−0.20; 1.14)	0.17	0.27	(0.02; 0.52)	0.03
Health (EQ‐5D‐5L)	−0.50	(−2.42; 1.41)	0.61	0.17	(−0.88; 0.55)	0.65	−0.08	(−1.69; 1.53)	0.92	−0.32	(−0.99; 0.36)	0.35
Education	0.02	(−0.10; 0,13)	0.79	0.04	(−0.01; 0.08)	0.14	0.03	(−0.14; 0.08)	0.59	0.02	(−0.03; 0.07)	0.36
Equivalized net income ≥ €2400	−0.48	(−1.14; 0.19)	0.16	−0.08	(−0.35; 0.18)	0.53	−0.30	(−0.91; 0.31)	0.34	0.19	(−0.06; 0.45)	0.14

*Note:* Joint‐log likelihood = −1124.03; BIC (Bayesian Information Criterion) = 2350.43 for WTP DIGA. Joint‐log likelihood = −1139.38; BIC (Bayesian Information Criterion) = 2380.63 for WTP DMP.

Abbreviations: Coeff = Coefficient, CI = Confidence Interval, GLM = General Linear Model.

The delta method was applied to assess the expected change in WTP per unit change in the covariates. For WTP for DiGA, a one‐unit increase in digital affinity was associated with an increase of €6.91 (*p* = 0.01), and each additional year of age was associated with an increase in WTP of €0.24 (*p* = 0.02). Participants who stated that they were female had a €4.35 higher WTP; however, this difference was not statistically significant (*p* = 0.05).

For WTP for DMP, a one‐unit increase in digital infrastructure was associated with an increase of €2.63 (*p* = 0.04), and female participants had an increase of €6.20 (*p* = 0.02) compared with male participants. Participants with an equivalized net income ≥ €2400 showed an estimated increase in WTP of €3.29, which was not statistically significant (*p* = 0.22). The delta method results are presented in Table 5.

**TABLE 5:** Estimated change in WTP.

	Marginal change in WTP					
	WTP for DiGA (*n* = 295)			WTP for DMP (*n* = 287)		
	Change (€)	(95% CI)	*p*‐values	Change (€)	(95% CI)	*p*‐values
Digital affinity (index)	6.91	(1.41; 12.40)	0.01	4.83	(−1.52; 11.18)	0.14
Medical infrastructure	0.26	(−1.76; 2.28)	0.80	0.30	(−2.08; 2.69)	0.80
Digital infrastructure	1.27	(−0.87; 3.41)	0.25	2.63	(0.11; 5.15)	0.04
Age	0.24	(0.04; 0.45)	0.02	−0.02	(−0.25; 0.22)	0.89
Sex (female)	4.35	(−0.08; 8.79)	0.05	6.20	(0.81; 11.60)	0.02
Equivalized net income ≥ €2400	−2.01	(−6.46; 2.44)	0.38	3.29	(−1.94; 8.51)	0.22
Education	0.62	(−0.21; 1.44)	0.14	0.41	(−0.58; 1.40)	0.42

Abbreviation: CI = Confidence Interval.

## 4 Discussion

This study found that individuals with a BMI of 30 kg/m^2^ or higher are willing to pay for weight loss programs if these services are not covered by statutory health insurance. The vast majority of respondents expressed a WTP, namely 95.9% for the DiGA and 95.5% for the DMP. Although the WTP for the DMP was slightly higher at €18.70 than for the DiGA at €16.15, this difference appears modest in substantive terms despite its statistical significance. Several explanations may account for this pattern. Respondents may have perceived the additional benefit of physician involvement as limited; an anchoring effect resulting from the order of the questions may have played a role. The expected clinical benefit (weight loss) seems to have been the decisive factor, rendering differences in the characteristics of the interventions less relevant. The finding that 176 of the 371 participants (47.4%) reported the same amount for both interventions supports the interpretation that the characteristics of the interventions were of only minor importance to many respondents. For the DMP, no amount of reimbursement is established yet, so that WTP cannot be compared to its costs. For the DiGA, the stated manufacturer prices per month, according to the DiGA directory, are approximately €70 (€218.00 for Zanadio, €220.90 for Oviva for 3 months), which is more than four times this amount.

Comparing these findings in the context of previous studies on WTP for overweight and obesity interventions in adults, one systematic review was identified [22]. Of the nine studies identified, four assessed monthly WTP. Liu et al. (2009) reported monthly WTP of approximately $12 for pharmacological treatment and $10 for a low‐calorie diet among employed women in Taiwan [25]. Doyle et al. (2012) reported WTP of £6.51/$10.49 per month per percentage point of weight loss for pharmacological interventions [26]. Jerome et al. reported a median monthly WTP of $45 for continuing the lifestyle‐based Hopkins POWER program [27]. Lauer et al. found a median monthly WTP of €20 for preventive programs targeting childhood overweight [28].

Overall, the magnitude of WTP observed in the study aligns well with this heterogeneous evidence base. The estimates are lower than those reported by Jerome et al. but close to the $10–$12 reported by Liu et al. and the €20 median reported by Lauer et al., despite differing preventive and treatment contexts. For comparison with Doyle et al., WTP estimates were converted to euros per month per percentage point of weight loss during the first 3 months (conversion based on the weight‐loss scenario presented in the questionnaire; see Supporting Information S1). This results in values of €5.31 for DiGA and €6.15 for DMP, which closely match the £6.51 or $10.49 reported by Doyle et al., even though their study evaluated a pharmacological rather than a behavioral intervention.

Another key finding is that 47.4% of respondents reported the same WTP for both interventions. In prior studies, such patterns have often been interpreted as scope insensitivity, meaning an insufficient differentiation between offerings of different scope or intensity [29, 30]. However, this explanation cannot be transferred to the present results because the two interventions did not differ in their expected clinical outcomes and varied only in their characteristics, such as the mode of delivery. The identical WTP values, therefore, do not represent a classical scope‐insensitive response but suggest that many respondents did not attribute substantial additional value to differences in delivery format. This interpretation aligns with the observation that expected clinical benefit was the decisive criterion for respondents [26].

The consistency of WTP across different interventions, target populations, and countries, along with the observation that 47.4% of respondents reported identical WTP for two intervention formats, may suggest a potential embedding effect. This means that respondents report their WTP for the overarching problem rather than for the specific intervention [31]. This effect has been repeatedly observed in the health sector [30, 32, 33].

With regard to the determinants of WTP, the regression analyses identified significant associations. Higher age and greater digital affinity were significantly associated with higher WTP for DiGAs. Contrary to previous studies, showing higher WTP among younger individuals for digital health or weight‐loss interventions [25, 34, 35], older participants in the study expressed higher WTP for digital interventions. This may reflect younger users' increased reliance on free apps versus older users' greater trust in certified medical products. Digital affinity was positively associated with WTP, supporting the assumption that individuals who are more open to digital technologies are also more willing to pay for digital health applications. Additionally, prior positive experiences with digital behavior‐change interventions may increase WTP. For the DMP, women showed significantly higher WTP than men, consistent with previous research [34, 36]. Two factors may explain this difference. First, women may tend to experience the psychosocial consequences of obesity more intensely than men, in part due to widespread stigmatization in areas such as education and employment [37, 38]. Second, women who experience weight‐related stigmatization may suffer more from the associated psychological burden than men in comparable situations [39, 40]. While the effect of sex on WTP for the DiGA was not statistically significant, the positive coefficient and comparable delta method magnitude suggest a similar directional pattern, indicating that sex‐related differences in the valuation of weight‐loss interventions may extend to both delivery modes, albeit more strongly for the DMP.

Digital infrastructure was significantly associated with higher WTP for the DMP. This finding appears counterintuitive, as a positive association was expected primarily for the DiGA, given that the use of digital applications depends on adequate internet access. A possible explanation is that the digital infrastructure variable captures broader contextual factors related to urbanization, encompassing aspects such as higher health awareness, stronger social comparison processes, greater exposure to health‐related risks, or shorter distances to healthcare facilities. In contrast to previous studies [22, 34, 41], health status (as measured by the EQ‐5D‐5L) had no significant impact on WTP. This may reflect that individuals with a BMI of 30 kg/m^2^ or higher may have experienced repeated unsuccessful attempts to lose weight, leading to skepticism regarding the effectiveness and sustainability of new interventions. Patients and healthcare professionals frequently perceive long‐term weight maintenance and the durability of weight loss effects as major barriers to participation in behavior‐based weight reduction programs [38]. Socioeconomic factors such as education and income also showed no significant influence on WTP. Evidence on socioeconomic characteristics is inconclusive, with some studies reporting positive associations [34, 36, 42] and others finding none [27, 35, 43].

Several limitations should be considered when interpreting the findings of this WTP analysis. The representativeness of the sample could have been improved with respect to key characteristics. In particular, higher‐income individuals were under‐represented, which may explain why the coefficients for income point in the expected direction but did not reach statistical significance. In addition, the sex distribution in the sample was skewed, with approximately 60% male participants, whereas among adults with obesity in Germany only 40% are male [34]. A further limitation is that preferences for a digital intervention were collected digitally. The digital survey made it possible not only to describe the app but also to visualize its structure and functions, thereby giving participants as concrete an idea as possible of the product being evaluated. At the same time, this may lead to a selection bias, as it under‐represents individuals who are less familiar with (and have less affinity for) digital media, and may thus hold a higher WTP for the DMP.

Additionally, participants received standard incentives from Forsa for completing the questionnaire, a common practice in survey research. While these incentives help ensure participation, they may also encourage faster responses, which could limit the time participants devote to reflecting on each question and providing fully considered WTP estimates. Furthermore, the analysis was restricted to WTP alone and did not involve a full cost‐benefit analysis. The comparison of WTP to prices ignores, for example, additional health care costs which may be incurred by the DiGA (e.g., reimbursement of physicians who prescribe the app) or DMP (e.g., costs of ongoing documentation and regular physician consultations) as well as any savings (e.g., avoided future healthcare costs associated with complications like diabetes).

Finally, access to reimbursement as DiGA involves high administrative costs in Germany. In general, public decision makers do not only have the option to decide whether or not to cover an intervention under given circumstances. Also, they can decide whether or not to modify the administrative burden so that interventions can be offered at lower prices. However, this was beyond the scope of this analysis as well.

Several implications for future research and practice emerge from these findings. The small differences in WTP between digital and offline formats, along with the high share of identical valuations, may indicate that respondents primarily value expected health gains rather than specific intervention characteristics. This raises a methodological question: frequently, theoretical papers stress the differences between WTP‐based cost‐benefit analyses and cost‐utility analyses. For this example of a digital obesity application, it would be worthwhile to assess whether the different evaluation approaches actually arrive at equivalent conclusions. A related question concerns the potential embedding effect in the context of obesity, which would be consistent with the results. Future research should explore potential determinants of this effect, for example, by varying intervention characteristics and expected weight loss outcomes. Moreover, given that both DiGA and DMP are novel interventions, future research should examine whether WTP evolves over time as more individuals gain experience with these programs. Motivation at enrollment and ongoing engagement could influence perceived value, but this remains to be empirically tested. Furthermore, the influence of participants' ability to pay on WTP remains ambiguous and was not significant in the study, warranting further investigation.

Participants' stated WTP (€16.15) was substantially lower than current reimbursement rates for certified digital health applications (approx. €70). In line with the paragraph above, future research should explore if this is a result of bias or misunderstanding (e.g., if respondents did not understand the difference between evidence‐based, privacy‐respecting digital care and apps without these characteristics available in app stores)—or, if it indicates that currently, German sickness funds reimburse services that are far from providing good value for money.

Finally, the decision of whether an intervention provides good value for money from the perspective of welfarist cost‐benefit analysis requires including all relevant costs and benefits so that a full health economic evaluation would be desirable to provide a better basis for such a decision.

From a practical and policy perspective, the findings suggest that the mode of delivery (digital vs. offline) plays a secondary role in shaping individuals' values of weight loss interventions, as long as expected effectiveness is comparable. This confirms the German practice of coverage and reimbursement decisions, which is guided primarily by evidence of clinical effectiveness, rather than assumptions about higher patient acceptance of digital formats.

Under the assumptions—which should be treated with caution—that, first, the survey responses were free of bias and the respondents were sufficiently well informed, and, second, that the omitted discounted expected savings due to avoided costs of obesity (e.g., avoided cases of diabetes) are lower than the omitted additional costs incurred by the apps (e.g., costs of physician visits as part of prescriptions), current DiGAs for obesity do not appear to provide good value for money from a welfarist cost‐benefit perspective.

## 5 Conclusion

Research on WTP for weight loss interventions is limited, particularly regarding digital formats. This study provides initial empirical evidence by comparing WTP for digital with offline interventions. Findings indicate that individuals with a BMI of 30 kg/m^2^ or higher show a WTP for weight reduction programs. The difference in WTP between digital and offline formats is statistically significant but moderate, suggesting that it is not the format but rather the perceived effectiveness of the intervention that exerts the strongest influence on WTP.

## Author Contributions

K.H. contributed to methodology (lead), writing – original draft, validation, visualization and writing – review and editing. S.K.L. contributed to the methodology, formal analysis, validation, and writing – review and editing. W.R. contributed to conceptualization, methodology, funding acquisition, and writing – review and editing. O.L. contributed to the methodology, project administration, supervision, and writing – review and editing. All authors approved the final manuscript.

## Funding

K.H. and O.L. partly received public funding from Leibniz Science Campus Bremen Digital Public Health (lsc‐diph.de), which is jointly funded by the Leibniz Association (W4/2018), the Federal State of Bremen and the Leibniz Institute for Prevention Research and Epidemiology—BIPS.

## Conflicts of Interest

The authors declare no conflicts of interest.

## Supporting information


Supporting Information S1

Supporting Information S2

## Data Availability

The data that support the findings of this study are available from the corresponding author upon reasonable request.

## References

[1] A. Schienkiewitz , R. Kuhnert , M. Blume , and G. Mensink , Übergewicht und Adipositas bei Erwachsenen in Deutschland‐Ergebnisse der Studie GEDA 2019/2020‐EHIS 2022, 10.25646/10292.

[2] T. Effertz , S. Engel , F. Verheyen , and R. Linder , “The Costs and Consequences of Obesity in Germany: A New Approach From a Prevalence and Life‐Cycle Perspective,” European Journal of Health Economics 17, no. 9 (2016): 1141–1158, 10.1007/s10198-015-0751-4. 26701837

[3] A. Konnopka , A. Dobroschke , T. Lehnert , and H.‐H. König , “Die Kosten von Übergewicht und Adipositas in Deutschland–ein systematischer Literaturüberblick,” Gesundheitswesen 80, no. 5 (2018): 471–481, 10.1055/s-0043-104692. 28561182

[4] K. Barnett , S. W. Mercer , M. Norbury , G. Watt , S. Wyke , and B. Guthrie , “Epidemiology of Multimorbidity and Implications for Health Care, Research, and Medical Education: A Cross‐Sectional Study,” Lancet 380, no. 9836 (2012): 37–43, 10.1016/s0140-6736(12)60240-2. 22579043

[5] A. Odone , S. Buttigieg , W. Ricciardi , N. Azzopardi‐Muscat , and A. Staines , “Public Health Digitalization in Europe: EUPHA Vision, Action and Role in Digital Public Health,” supplement, European Journal of Public Health 29, no. S3 (2019): 28–35, 10.1093/eurpub/ckz161. 31738441 PMC6859512

[6] A. L. Stark , C. Geukes , and C. Dockweiler , “Digital Health Promotion and Prevention in Settings: Scoping Review,” Journal of Medical Internet Research 24, no. 1 (2022): e21063, 10.2196/21063. 35089140 PMC8838600

[7] O. Lange , “Health Economic Evaluation of Preventive Digital Public Health Interventions Using Decision‐Analytic Modelling: A Systematized Review,” BMC Health Services Research 23, no. 1 (2023): 268, 10.1186/s12913-023-09280-3. 36932436 PMC10024449

[8] M. L. Patel , L. N. Wakayama , and G. G. Bennett , “Self‐Monitoring via Digital Health in Weight Loss Interventions: A Systematic Review Among Adults With Overweight or Obesity,” Obesity 29, no. 3 (2021): 478–499, 10.1002/oby.23088. 33624440 PMC12838191

[9] K. Liu , Z. Xie , and C. K. Or , “Effectiveness of Mobile App‐Assisted Self‐Care Interventions for Improving Patient Outcomes in Type 2 Diabetes and/or Hypertension: Systematic Review and Meta‐Analysis of Randomized Controlled Trials,” JMIR mHealth and uHealth 8 (2020): e15779, 10.2196/15779. 32459654 PMC7435643

[10] S. Wongvibulsin , E. E. Habeos , P. P. Huynh , et al., “Digital Health Interventions for Cardiac Rehabilitation: Systematic Literature Review,” Journal of Medical Internet Research 23, no. 2 (2021): e18773, 10.2196/18773. 33555259 PMC7899799

[11] B. Marques , J. McIntosh , A. Valera , and A. Gaddam , “Innovative and Assistive Ehealth Technologies for Smart Therapeutic and Rehabilitation Outdoor Spaces for the Elderly Demographic,” Multimodal Technologies and Interaction 4 (2020): 76, 10.3390/mti4040076.

[12] N. M. Aashima and R. Sharma , “A Review of Patient Satisfaction and Experience With Telemedicine: A Virtual Solution During and Beyond COVID‐19 Pandemic,” Telemed E‐Health 27 (2021): 1325–1331, 10.1089/tmj.2020.0570. 33719577

[13] M. O’Cathail , M. A. Sivanandan , C. Diver , P. Patel , and J. Christian , “The Use of Patient‐Facing Teleconsultations in the National Health Service: Scoping Review,” JMIR Medical Informatics 8, no. 3 (2020): e15380, 10.2196/15380. 32175911 PMC7105931

[14] A. J. Dawes , A. Y. Lin , C. Varghese , M. M. Russell , and A. Y. Lin , “Mobile Health Technology for Remote Home Monitoring After Surgery: A Meta‐Analysis,” British Journal of Surgery 108, no. 11 (2021): 1304–1314, 10.1093/bjs/znab323. 34661649

[15] M. Peyroteo , I. A. Ferreira , L. B. Elvas , J. C. Ferreira , and L. V. Lapão , “Remote Monitoring Systems for Patients With Chronic Diseases in Primary Health Care: Systematic Review,” JMIR mHealth and uHealth 9, no. 12 (2021): e28285, 10.2196/28285. 34932000 PMC8734917

[16] M. Uncovska , B. Freitag , S. Meister , and L. Fehring , “Patient Acceptance of Prescribed and Fully Reimbursed mHealth Apps in Germany: An UTAUT2‐Based Online Survey Study,” Journal of Medical Systems 47, no. 1 (2023): 14, 10.1007/s10916-023-01910-x. 36705853 PMC9880914

[17] Bundestag , Gesetz für eine bessere Versorgung durch Digitalisierung und Innovation (Digitale‐Versorgung‐Gesetz – DVG), Vol. 49 (Bundesgesetzblatt, 2019).

[18] G. Bundesausschuss . Beschluss des Gemeinsamen Bundesausschusses über die 34. Änderung der DMP‐Anforderungen‐Richtlinie (DMP‐A‐RL): Änderung der Anlage 2, Ergänzung der Anlage 23 (DMP Adipositas) und der Anlage 24 (Adipositas Dokumentation) 2023.

[19] Statista , Anzahl der bei Google Play verfügbaren mHealth‐Apps, (2025).

[20] E. McIntosh , Applied Methods of Cost‐Benefit Analysis in Health Care (Oxford University Press, 2010).

[21] C. Steigenberger , M. Flatscher‐Thoeni , U. Siebert , and A. M. Leiter , “Determinants of Willingness to Pay for Health Services: A Systematic Review of Contingent Valuation Studies,” European Journal of Health Economics 23, no. 9 (2022): 1455–1482, 10.1007/s10198-022-01437-x. PMC8853086 35166973

[22] N. A. Anwar , A. N. Aizuddin , N. Ahmad , A. Aziz , and N. A. Anwar , “Attributes Influencing Willingness to Pay for Overweight and Obesity Interventions in Adults: A Systematic Literature Review,” Cureus 16 (2024), 10.7759/cureus.65468. PMC11281942 39071073

[23] K. Ludwig , J.‐M. Graf von der Schulenburg , and W. Greiner , “German Value Set for the EQ‐5D‐5L,” PharmacoEconomics 36, no. 6 (2018): 663–674, 10.1007/s40273-018-0615-8. 29460066 PMC5954069

[24] P. Deb , E. C. Norton , and W. G. Manning , Health Econometrics Using Stata, Vol. 3 (Stata press, 2017).

[25] J.‐T. Liu , M.‐W. Tsou , and J. K. Hammitt , “Willingness to Pay for Weight‐Control Treatment,” Health Policy 91, no. 2 (2009): 211–218, 10.1016/j.healthpol.2008.12.011. 19167128

[26] S. Doyle , A. Lloyd , J. Birt , et al., “Willingness to Pay for Obesity Pharmacotherapy,” Obesity 20, no. 10 (2012): 2019–2026, 10.1038/oby.2011.387. 22301901

[27] G. J. Jerome , R. Alavi , G. L. Daumit , et al., “Willingness to Pay for Continued Delivery of a Lifestyle‐Based Weight Loss Program: The Hopkins POWER Trial,” Obesity 23, no. 2 (2015): 282–285, 10.1002/oby.20981. 25557807 PMC4310798

[28] R. Lauer , M. Traub , S. Hansen , R. Kilian , J. M. Steinacker , and D. Kesztyüs , “Longitudinal Changes and Determinants of Parental Willingness to Pay for the Prevention of Childhood Overweight and Obesity,” Health Economics Review 10, no. 1 (2020): 15, 10.1186/s13561-020-00266-z. 32468490 PMC7257510

[29] K. L. Giraud , J. B. Loomis , and R. L. Johnson , “Internal and External Scope in Willingness‐to‐Pay Estimates for Threatened and Endangered Wildlife,” Journal of Environmental Management 56, no. 3 (1999): 221–229, 10.1006/jema.1999.0277.

[30] D. Gyrd‐Hansen , T. Kjær , and J. S. Nielsen , “Scope Insensitivity in Contingent Valuation Studies of Health Care Services: Should We Ask Twice?,” Health Economics 21, no. 2 (2012): 101–112, 10.1002/hec.1690. 22223555

[31] D. Kahneman and J. L. Knetsch , “Valuing Public Goods: The Purchase of Moral Satisfaction,” Journal of Environmental Economics and Management 22, no. 1 (1992): 57–70, 10.1016/0095-0696(92)90019-s.

[32] A. Shiell and L. Gold , “Contingent Valuation in Health Care and the Persistence of Embedding Effects Without the Warm Glow,” Journal of Economic Psychology 23, no. 2 (2002): 251–262, 10.1016/s0167-4870(02)00066-1.

[33] J. A. Olsen , C. Donaldson , and J. Pereira , “The Insensitivity of Willingness‐to‐Pay to the Size of the Good: New Evidence for Health Care,” Journal of Economic Psychology 25, no. 4 (2004): 445–460, 10.1016/s0167-4870(03)00029-1.

[34] K. Narbro and L. Sjöström , “Willingness to Pay for Obesity Treatment,” International Journal of Technology Assessment in Health Care 16, no. 1 (2000): 50–59, 10.1017/s0266462300016159. 10815353

[35] C. Somers , E. Grieve , M. Lennon , M.‐M. Bouamrane , F. S. Mair , and E. McIntosh , “Valuing Mobile Health: An Open‐Ended Contingent Valuation Survey of a National Digital Health Program,” JMIR mHealth and uHealth 7, no. 1 (2019): e9990, 10.2196/mhealth.9990. PMC6354197 30664488

[36] T.‐T. Fu , Y.‐M. Lin , and C. L. Huang , “Willingness to Pay for Obesity Prevention,” Economics and Human Biology 9, no. 3 (2011): 316–324, 10.1016/j.ehb.2011.02.003. 21497145

[37] C. Anderson , C. B. Peterson , L. Fletcher , J. E. Mitchell , P. Thuras , and S. J. Crow , “Weight Loss and Gender: An Examination of Physician Attitudes,” Obesity Research 9, no. 4 (2001): 257–263, 10.1038/oby.2001.30. 11331429

[38] M. V. Roehling , “Weight‐Based Discrimination in Employment: Psychological and Legal Aspects,” Personnel Psychology 52, no. 4 (1999): 969–1016, 10.1111/j.1744-6570.1999.tb00186.x.

[39] R. M. Puhl , T. Andreyeva , and K. D. Brownell , “Perceptions of Weight Discrimination: Prevalence and Comparison to Race and Gender Discrimination in America,” International Journal of Obesity 32, no. 6 (2008): 992–1000, 10.1038/ijo.2008.22. 18317471

[40] A. N. Fabricatore and T. A. Wadden , “Psychological Aspects of Obesity,” Clinical Dermatology 22, no. 4 (2004): 332–337, 10.1016/j.clindermatol.2004.01.006. 15475235

[41] Å Romé , U. Persson , C. Ekdahl , and G. Gard , “Willingness to Pay for Health Improvements of Physical Activity on Prescription,” Scandinavian Journal of Public Health 38, no. 2 (2010): 151–159, 10.1177/1403494809357099. 20064920

[42] C. Khawali , M. B. Ferraz , M. T. Zanella , and S. R. Ferreira , “Willingness to Pay as Patient Preference to Bariatric Surgery,” Health Expectations 17, no. 1 (2014): 73–81, 10.1111/j.1369-7625.2011.00738.x. 22070389 PMC5060693

[43] A. Wiesemann , U. Mueller‐Buehl , R. Scheidt , W. Boehme , and W. Scheuermann , “Patient Willingness to Pay for Preventive Measures in Primary Care: A Study of Five GPs in a German Community,” Social and Preventive Medicine 49, no. 4 (2004): 254–260, 10.1007/s00038-004-3039-5. 15357527

