# Supplementary material for: Does Digital Make a Difference? Willingness‐to‐Pay for Digital Versus Offline Weight Loss in Germany
Source: Obes Sci Pract. 2026 May 21;12(3):e70151. doi: 10.1002/osp4.70151 (PMC13240293; doi:10.1002/osp4.70151)
Supplement: Supplementary file 1 — Supporting Information S1 [file OSP4-12-e70151-s002.pdf]

Supporting information 1: The full questionnaire

Summary: This file contains the full online questionnaire used in the contingent valuation survey. It includes all questions on sociodemographic characteristics, health status, digital affinity, and obesity-related experiences, as well as detailed descriptions of the intervention scenarios (DiGA and DMP) and the willingness-to-pay elicitation using a payment-scale format.

Intro

Einleitung: Herzlich Willkommen zu unserer Umfrage zum Thema Apps und Gesundheit.

Alle Antworten werden anonymisiert. Bitte antworten Sie so offen wie möglich, es gibt keine richtigen oder falschen Antworten.

Apps sind Anwendungen auf mobilen Endgeräten (z. B. Smartphones oder Tablets). Es gibt sowohl kostenlose als auch kostenpflichtige Apps.

Frage 1a

Nutzen Sie eine oder mehrere Apps oder haben Sie bereits Apps genutzt?

1. Ja
2. Nein
3. Keine Angabe

<u>Filter: Nutzen Apps oder haben bereits Apps genutzt oder k.A (Frage 1=1 oder 3)</u>

Frage 1b

Wie ist Ihre Einstellung gegenüber Apps?

1. Sehr positiv
2. Positiv
3. Neutral
4. Negativ
5. Sehr negativ

<u>Filter: Nutzen Apps oder haben bereits Apps genutzt (Frage 1=1 oder 3)</u>

Frage 1c

Wie bewerten Sie ihre eigenen Kenntnisse im Umgang mit Apps?

1. Sehr gut
2. Gut
3. Neutral
4. Schlecht
5. Sehr schlecht

Filter: Nutzen Apps oder haben bereits Apps genutzt oder k.A (Frage 1=1 oder 3))

Frage 2

Haben Sie bereits für die Nutzung einer App Geld bezahlt?

1. Ja
2. Nein
3. Keine Angabe

Filter: Nutzen Apps oder haben bereits Apps genutzt oder k.A. (Frage 1=1 oder 3))

Frage 3

Apps, die Funktionalitäten für die Bereiche Gesundheit, Medizin, Heilkunde oder Wellness bereitstellen, werden als Gesundheitsapps bezeichnet. Gesundheitsapps stehen im Fokus dieser Umfrage.

Nutzen Sie eine oder mehrere Gesundheitsapps oder haben Sie bereits Gesundheitsapps genutzt?

1. Ja
2. Nein
3. Keine Angabe

Filter: Nutzen Gesundheitsapps (Frage 3=1)

Frage 4

Haben Sie bereits für die Nutzung einer Gesundheitsapp Geld bezahlt?

1. Ja
2. Nein
3. Keine Angabe

Frage 5

Viele Gesundheits-Apps befassen sich mit den Schwerpunkten Ernährung, Bewegung und Körpergewicht.

Wie ist Ihre Körpergröße in Zentimetern?

1. [NUMERISCHEEINGABE]
2. Keine Angabe

Frage 6

Wie ist Ihr Körpergewicht in Kilogramm?

1. [NUMERISCHEEINGABE]
2. Keine Angabe

Frage 7

Seit 2019 können in Deutschland bestimmte Apps von Ärzten und Ärztinnen auf Rezept verschrieben werden. Diese Apps werden digitale Gesundheitsanwendungen (kurz DiGA) genannt. Wird eine DiGA auf Rezept verschrieben, werden die Kosten von der Krankenkasse übernommen.

Haben Sie Erfahrungen mit DiGAs, sei es durch eigene Nutzung oder Nutzung im Familien-, Freundes- oder Bekanntenkreis?

1. Ja eigene Nutzung
2. Ja Nutzung im Familien-, Freundes- oder Bekanntenkreis
3. Nein
4. Keine Angabe

Intro

Im Gegensatz zu frei verfügbaren Gesundheitsapps verfügen DiGAs über eine offizielle Zulassung durch das Bundesinstitut für Arzneimittel und Medizinprodukte (BfArM). Für diese Zulassung muss sichergestellt sein, dass die App dem aktuellen Stand medizinischer Forschung entspricht und besonders hohe Sicherheits- und Datenschutzstandards einhält. Zudem muss ihre Wirksamkeit mit klinischen Studien wissenschaftlich belegt werden.

DiGAs haben das Ziel, Menschen bei der Bewältigung von Krankheiten und der Verbesserung der Lebensqualität zu unterstützen. Dabei können sie medizinischen Nutzen haben, wie die Erkennung, Überwachung, Behandlung oder Linderung von Krankheiten. Zudem können sie Strukturen verbessern, z.B. durch einen einfacheren Zugang zur Gesundheitsversorgung oder eine bessere Bewältigung des Alltags trotz Krankheit.

Frage 8

Wie ist Ihre Meinung zu DiGAs?

1. Positiv
2. Neutral
3. Negativ
4. Keine Angabe

<u>Filter: Keine eigene Nutzung von DiGAs (Frage 7=2,3 oder 4)</u>

Frage 9

Können Sie sich vorstellen, selbst DiGAs zu nutzen?

1. Ja
2. Nein
3. Keine Angabe

Frage 10

Sollten DiGAs von der gesetzlichen Krankenkasse finanziert werden (wie es derzeit der Fall ist)?

1. Ja
2. Nein
3. Keine Angabe

Frage 11

Wie bewerten Sie die Erreichbarkeit von Ärzten und Ärztinnen und anderen Gesundheitsleistungen an Ihrem Wohnort?

1. Sehr schlecht
2. Schlecht
3. Neutral
4. Gut
5. Sehr gut
6. Keine Angabe

Frage 12

Wie bewerten Sie die Internetverbindung an den Orten, an denen Sie sich im Alltag am häufigsten aufhalten? Denken Sie dabei auch an die Wege zwischen den verschiedenen Orten.

1. Sehr schlecht
2. Schlecht
3. Neutral
4. Gut
5. Sehr gut
6. Keine Angabe

Intro

Bitte lesen Sie die nachfolgenden Informationen sorgfältig durch.

Ein Gesundheitsproblem, für das DiGAs entwickelt wurden, ist Adipositas. Adipositas ist eine chronische Krankheit, die definiert ist als eine über das Normalmaß hinausgehende Vermehrung des Körperfetts. Wichtig für die Diagnose ist der Körpermasseindex, der sogenannte Body Mass Index (BMI). Der BMI setzt die Körpergröße und das Körpergewicht in ein Verhältnis. Ab einem BMI von 30 kann man von Adipositas sprechen. Etwa 19 Prozent der erwachsenen Bevölkerung in Deutschland sind davon betroffen, was ungefähr 13 Millionen Menschen entspricht.

Unterschiedliche Ursachen können die Entstehung von Adipositas fördern, wie beispielsweise Lebensstil, genetische Faktoren, Umweltbedingungen oder bestimmte Medikamente. Adipositas senkt die Lebenserwartung und erhöht das Risiko für Erkrankungen wie Typ-2-Diabetes, Herz-Kreislauf-Erkrankungen, verschiedene Krebsarten sowie Muskel- und Skeletterkrankungen. Menschen mit Adipositas erfahren oft Einschränkungen und sind häufig Stigmatisierung ausgesetzt, was mit psychischen Erkrankungen wie Depressionen einhergehen kann.

Frage 13

Haben Sie persönliche Erfahrungen mit Adipositas, zum Beispiel über Betroffene im Familien- oder Freundeskreis?

1. Ja
2. Nein

3. Keine Angabe

<u>Filter: BMI unter 30 (Frage 5 und 6 = BMI unter 30)</u>

Frage 14

Wie hoch schätzen Sie Ihr persönliches Risiko ein, an Adipositas zu erkranken?

1. Kein Risiko
2. Sehr gering
3. Gering
4. Mittel
5. Hoch
6. Sehr hoch
7. Keine Angabe

<u>Filter: BMI über 30 (Frage 5 und 6 = BMI über 30)</u>

Frage 15

Erleben Sie derzeit Einschränkungen/Beschwerden aufgrund Ihres Körpergewichts?

1. Keine Einschränkungen/Beschwerden
2. Leichte Einschränkungen/Beschwerden
3. Mäßige Einschränkungen/Beschwerden
4. Starke Einschränkungen/Beschwerden
5. Extreme Einschränkungen/Beschwerden
6. Keine Angabe

Intro

Die Zulassung durch das *Bundesinstitut für Arzneimittel und Medizinprodukte* (BfArM) als DiGAs zur Adipositas-Behandlung haben die Apps Zanadio und Oviva erhalten. Diese Apps zielen darauf ab, das Gewicht der Patienten und Patientinnen zu reduzieren, das reduzierte Gewicht langfristig zu halten und die Gesundheit sowie das Wohlbefinden zu verbessern.

Frage 16

Haben Sie Erfahrungen mit der Nutzung von Zanadio oder Oviva (eigene Nutzung oder Nutzung im Familien-, Freundes- oder Bekanntenkreis)?

1. Ja, eigene Erfahrung mit Zanadio oder Oviva
2. Ja, Erfahrung mit Zanadio oder Oviva im Familien-, Freundes- oder Bekanntenkreis
3. Nein
4. Keine Angabe

Intro

Bitte lesen Sie die nachfolgenden Informationen sorgfältig durch und nehmen sich die Zeit, die Bilder anzuschauen.

Um eine langfristige Gewichtsreduktion zu ermöglichen, setzen Zanadio und Oviva auf die Umstellung der Ernährung, die Förderung der alltäglichen Bewegung und Verhaltensänderungen. Beide Apps verfügen über folgende Funktionen:

Mit einem Klick auf das jeweilige Bild können Sie dieses vergrößern.

- Tagebuchfunktion zur Erfassung der Mahlzeiten und zur Erfassung der körperlichen Aktivitäten (manuell oder automatisch über Fitnesstracker, Smartwatch o.ä.).
  [Abbildung hier nicht abrufbar]

- Umfassende Informationen darüber, wie Ernährung, Bewegung und Verhalten das Gewicht beeinflussen. Diese Informationen werden über Videos und kurze Texte vermittelt.
  [Abbildung hier nicht abrufbar]

- Motivationsunterstützung, z.B. durch individuelle Zielsetzungen und der Darstellung von Ergebnissen in verschiedenen Grafiken.
  [Abbildung hier nicht abrufbar]

- Chatfunktion, mit der Nutzer und Nutzerinnen eine Ernährungsberatung erhalten oder weitere Themen und Fragen klären können. Entsprechend der gesetzlichen Verpflichtung bieten alle DiGAs einen technischen Support an, der innerhalb von 24 Stunden Rückmeldungen gibt.
  [Abbildung hier nicht abrufbar]

Intro

Ein adipöser Mann mit einer Körpergröße von 180 cm und einem Körpergewicht von 115 kg könnte durch Nutzung der DiGAs in drei Monaten durchschnittlich 3,5 Kilogramm und in 12 Monaten durchschnittlich 9,2 Kilogramm abnehmen. Diese Auswirkungen auf die Gewichtsveränderung sind ähnlich oder sogar stärker als bei klassischen analogen Adipositas-Therapien.

Obwohl eine derartige Gewichtsreduktion noch keine Heilung der Adipositas bedeutet, sind mit der Verringerung von Übergewicht positive Effekte verbunden. Somit trägt die Gewichtsreduktion zur Lebenserwartung bei, reduziert das Risiko von Folgeerkrankungen wie beispielsweise Krebs, Diabetes oder Herz-Kreislauf-Erkrankungen und kann zu einer Verbesserung psychischer Erkrankungen wie zum Beispiel Depressionen führen.

Im Folgenden möchten wir herausfinden, wie viel Geld Sie für eine DiGA zur Adipositas-Behandlung maximal bezahlen würden. Gehen Sie dabei von einer DiGA aus, die in ihren Funktionen der Beschreibung von Zanadio und Oviva entspricht. Die DiGA wird für drei Monate erworben und endet automatisch. Bei Bedarf kann die Laufzeit verlängert werden.

Frage 17

Filter: BMI über 30 (Frage 5 und 6 = BMI über 30)

Stellen Sie sich eine Situation vor, in der die DiGA nicht von der Krankenkasse finanziert wird. Wie viel Euro wären Sie maximal bereit, pro Monat für eine DiGA zur Adipositas-Behandlung zu bezahlen?

Filter: BMI unter 30 (Frage 5 und 6 = BMI unter 30)

Stellen Sie sich eine Situation vor, in der Sie an Adipositas erkrankt sind und die DiGA nicht von der Krankenkasse finanziert wird. Wie viel Euro wären Sie maximal bereit, pro Monat für eine DiGA zur Adipositas-Behandlung zu bezahlen?

[Die Probanden werden den drei unterschiedlichen Zahlungskarten zufällig zugelost.]

Bitte betrachten Sie die Werte der Tabelle. **Markieren Sie alle Werte**, die Sie auf jeden Fall für die DiGA bezahlen würden. Beginnen Sie mit dem niedrigsten Wert und hören Sie mit dem höchsten Wert auf, den Sie zu zahlen bereit sind. Bitte markieren Sie keinen Wert, bei dem Sie sich unsicher sind.

Skala 1	Der monatliche Betrag für die DiGA zur Adipositas-Behandlung den ich definitiv zahlen würde, ist
0 €	□
1 €	□
3 €	□
5 €	□
7 €	□
10 €	□
15 €	□
30 €	□
50 €	□
75 €	□
100 €	□
Mehr als 100 €	□

Keine Angabe

Skala 2	Der monatliche Betrag für die DiGA zur Adipositas-Behandlung den ich definitiv zahlen würde, ist
0 €	□
5 €	□
10 €	□
20 €	□

30 €	◻
40 €	◻
50 €	◻
60 €	◻
70 €	◻
80 €	◻
100 €	◻
Mehr als 100 €	◻

Keine Angabe

Skala 3	Der monatliche Betrag für die DiGA zur Adipositas-Behandlung den ich definitiv zahlen würde, ist
0 €	◻
5 €	◻
10 €	◻
20 €	◻
30 €	◻
40 €	◻
80 €	◻
120 €	◻
200 €	◻
300 €	◻
400 €	◻
Mehr als 400 €	◻

Keine Angabe

Filter: Zahlungsbereitschaft größer als Werte in der Tabelle (Frage 17: Mehr als 100 und Mehr als 400)

Frage 18

Wie viel wären Sie definitiv bereit, monatlich zu zahlen?

1. [Eingabefeld]
2. Keine Angabe

Frage 19(1)

Welcher Geldbetrag pro Monat wäre Ihnen für die DiGA definitiv zu hoch?

[Die Probanden erhalten die gleiche Skala wie bei der vorhergehenden Frage]

Bitte betrachten Sie nochmals die Werte der Tabelle. **Markieren Sie alle Werte**, die Sie auf keinen Fall für die DiGA bezahlen würden. Beginnen Sie mit dem höchsten Wert und hören Sie mit dem niedrigsten Wert auf, den Sie nicht zahlen würden. Bitte markieren Sie keinen Wert, bei dem Sie unsicher sind.

Skala 1 B	Der monatliche Betrag für die DiGA zur Adipositas-Behandlung den ich definitiv **nicht** zahlen würde, ist
Der Betrag ist größer als 100 €	□
100€	□
75€	□
50€	□
30€	□
15€	□
10€	□
7€	□
5€	□
3€	□
1€	□
0€	□

Keine Angabe

Skala 2 B	Der monatliche Betrag für die DiGA zur Adipositas-Behandlung den ich definitiv **nicht** zahlen würde, ist
Der Betrag ist größer als 100 €	□
100€	□
80€	□
70€	□
60€	□
50€	□
40€	□
30€	□
20 €	□
10 €	□
5 €	□
0€	□

Keine Angabe

Skala 3B	Der monatliche Betrag für die DiGA zur Adipositas-Behandlung den ich definitiv **nicht** zahlen würde, ist
Der Betrag ist größer als 400 €	□
400€	□
300€	□
200€	□
120€	□
80€	□
40€	□
30€	□
20€	□
10€	□
5€	□
0€	□

Keine Angabe

Filter: Wenn in Frage 19(1): nur „größer als 100“ oder „größer als 400“ eingegeben wurde

Frage 19(2)

Welcher Geldbetrag pro Monat wäre Ihnen für die DiGA definitiv zu hoch?

1. [Eingabefeld]
2. Keine Angabe

Intro

Zur Behandlung von chronischen Erkrankungen stehen neben DiGAs auch strukturierte Behandlungsprogramme zur Verfügung, die „Disease-Management-Programme“ (DMP) genannt werden. DMPs fördern eine strukturierte Behandlung auf dem aktuellen Stand medizinischer Forschung. Es sind Programme für hausärztliche Praxen, in die auch Fachärzte und Fachärztinnen mit einbezogen werden. Im November 2023 wurde die Einrichtung eines DMP für Adipositas beschlossen.

Ebenso wie die digitale Adipositas-Therapie hat das analoge DMP für Adipositas das Ziel, durch Verhaltensänderungen eine dauerhafte Gewichtsreduktion und -stabilisierung zu erreichen. Hierfür erhalten DMP-Teilnehmende von ihrem Hausarzt bzw. ihrer Hausärztin individualisierte Empfehlungen für Ernährungs-, Bewegungs- und Verhaltensänderungen und Zugang zu einem Schulungsprogramm. Im Gegensatz zu DiGAs erfolgen die Betreuung der DMP-Teilnehmenden und die Dokumentation nicht digital und jederzeit verfügbar über eine App, sondern persönlich bei regelmäßigen Terminen (z.B. quartalsweise) in der hausärztlichen Praxis.

Frage 20

Anstelle der Bezeichnung "DMP" werden die strukturierten Behandlungsprogramme teilweise auch Curaplan genannt. Diese strukturierten Behandlungsprogramme existieren für verschiedene chronische Erkrankungen, wie

beispielsweise Asthma bronchiale, Brustkrebs, Herzinsuffizienz, COPD, Rückenschmerzen oder Diabetes mellitus Typ 1 und Typ 2.

Haben Sie Erfahrungen mit DMPs, sei es durch eigene Nutzung oder Nutzung im Familien-, Freundes- oder Bekanntenkreis?

1. Ja eigene Nutzung
2. Ja Nutzung im Familien-, Freundes- oder Bekanntenkreis
3. Nein
4. Keine Angabe

Intro

Im Folgenden möchten wir herausfinden, wie viel Geld Sie für die Teilnahme an einer DMP zur Adipositas-Behandlung maximal bezahlen würden. Gehen Sie dabei davon aus, dass das DMP in seiner Wirksamkeit vergleichbar mit der DiGA zur Adipositas-Therapie ist. Berücksichtigen Sie die Vorteile des DMP, wie bspw. den persönlichen Kontakt aber auch die Nachteile des DMP wie bspw. den erhöhten Zeitaufwand durch Anfahrtswege oder Wartezeiten.

<u>Frage 20(1)</u>

<u>Filter: BMI über 30 (Frage 5 und 6 = BMI über 30)</u>

Stellen Sie sich eine Situation vor, in der das DMP nicht von der Krankenkasse finanziert wird. Wie viel Euro wären Sie maximal bereit, pro Monat für die Teilnahme an dem DMP zur Adipositas-Behandlung zu bezahlen?

<u>Filter: BMI unter 30 (Frage 5 und 6 = BMI unter 30)</u>

Stellen Sie sich eine Situation vor, in der Sie an Adipositas erkrankt sind und das DMP nicht von der Krankenkasse finanziert wird. Wie viel Euro wären Sie maximal bereit, pro Monat für die Teilnahme an dem DMP zur Adipositas-Behandlung zu bezahlen?

Skala: Payment-Karte

[Die Probanden erhalten die gleichen Zahlungskarten wie zuvor.]

Bitte betrachten Sie die Werte der Tabelle. **Markieren Sie <u>alle Werte</u>**, die Sie <u>auf jeden Fall</u> für die Teilnahme an dem DMP <u>bezahlen</u> würden. Beginnen Sie mit dem niedrigsten Wert und hören Sie mit dem höchsten Wert auf, den Sie zu zahlen bereit sind. Bitte markieren Sie keinen Wert, bei dem Sie sich unsicher sind.

Skala 1	Der monatliche Betrag für die Teilnahme an dem DMP für Adipositas, den ich definitiv zahlen würde, ist
0 €	□
1 €	□

3 €	□
5 €	□
7 €	□
10 €	□
15 €	□
30 €	□
50 €	□
75 €	□
100 €	□
Mehr als 100 €	□

Keine Angabe

Skala 2	Der monatliche Betrag für die Teilnahme an dem DMP für Adipositas, den ich definitiv zahlen würde, ist
0 €	□
5 €	□
10 €	□
20 €	□
30 €	□
40 €	□
50 €	□
60 €	□
70 €	□
80 €	□
100 €	□
Mehr als 100 €	□

Keine Angabe

Skala 3	Der monatliche Betrag für die Teilnahme an dem DMP für Adipositas, den ich definitiv zahlen würde, ist
0 €	□
5 €	□
10 €	□
20 €	□
30 €	□

40 €	□
80 €	□
120 €	□
200 €	□
300 €	□
400 €	□
Mehr als 400 €	□

Keine Angabe

Filter: Zahlungsbereitschaft größer als Werte in der Tabelle (Frage 20(1): Mehr als 100 und Mehr als 400)

Frage 20(2)

Wie viel wären Sie definitiv bereit, monatlich zu zahlen?

1. [NUMERISCHEEINGABE]
2. Keine Angabe

Frage 20(3)

Welcher Geldbetrag pro Monat wäre Ihnen für die Teilnahme an dem DMP definitiv zu hoch?

Bitte betrachten Sie nochmals die Werte der Tabelle. **Markieren Sie alle Werte**, die Sie auf keinen Fall für die Teilnahme an dem DMP für Adipositas bezahlen würden. Beginnen Sie mit dem höchsten Wert und hören Sie mit dem niedrigsten Wert auf, den Sie nicht zahlen würden. Bitte markieren Sie keinen Wert, bei dem Sie unsicher sind.

[Die Probanden erhalten die gleiche Skala wie bei der vorhergehenden Frage zur DiGA]

Skala 1 B	Der monatliche Betrag für die Teilnahme an dem DMP für Adipositas den ich definitiv **nicht** zahlen würde, ist
Der Betrag ist größer als 100 €	□
100€	□
75€	□
50€	□
30€	□
15€	□
10€	□
7€	□
5€	□
3€	□

1€	◻
0€	◻

Keine Angabe

Skala 2 B	Der monatliche Betrag für die Teilnahme an dem DMP für Adipositas den ich definitiv **nicht** zahlen würde, ist
Der Betrag ist größer als 100 €	◻
100€	◻
80€	◻
70€	◻
60€	◻
50€	◻
40€	◻
30€	◻
20 €	◻
10 €	◻
5 €	◻
0€	◻

Keine Angabe

Skala 3B	Der monatliche Betrag für die Teilnahme an dem DMP für Adipositas den ich definitiv **nicht** zahlen würde, ist
Der Betrag ist größer als 400 €	◻
400€	◻
300€	◻
200€	◻
120€	◻
80€	◻
40€	◻
30€	◻
20€	◻
10€	◻
5€	◻
0€	◻

Keine Angabe

Filter: Zahlungsbereitschaft größer als Werte in der Tabelle (Frage 20(1): größer als 100 und größer als 400)

Frage 20(4)

Welcher Geldbetrag pro Monat wäre Ihnen für die Teilnahme an dem DMP definitiv zu hoch?

1. [NUMERISCHEEINGABE]
2. Keine Angabe

Frage 20(5)

Sie haben angegeben, dass Ihre maximale Zahlungsbereitschaft für eine DiGA zur Adipositas-Therapie zwischen [Wert aus Frage 17/18] und [Wert auf Frage 19] Euro liegt.

Ihre angegebene maximale Zahlungsbereitschaft für die Teilnahme an dem DMP für Adipositas liegt zwischen [Wert aus Frage20(1)/20(2)] und [Wert auf Frage 20(3)] Euro.

Ist dies realistisch oder möchten Sie Ihre Eingaben korrigieren?

1. Beide Angaben sind richtig
2. Zahlungsbereitschaft DIGA korrigieren
3. Zahlungsbereitschaft DMP korrigieren

[Nach der Korrektur erneut zu Frage 20(5), wobei die Werte als neue Variable separat erfasst werden]

Frage 21

Was war Ihnen bei der Bestimmung der Zahlungsbereitschaften besonders wichtig? Was waren die ausschlaggebenden Gründe für Ihre Entscheidungen?

In Hinblick auf die DiGA:
[Eingabefeld für offene Frage]

In Hinblick auf das DMP:
[Eingabefeld für offene Frage]

Frage 22

In welchem Jahr sind Sie geboren?

1. [NUMERISCHEEINGABE]
2. Keine Angabe

Frage 23

Geschlecht des Befragten

1. Männlich

2. Weiblich
3. Divers
4. Keine Angabe

Frage 24

Sind Sie zurzeit erwerbstätig? Unter Erwerbstätigkeit wird jede bezahlte bzw. mit einem Einkommen verbundene Tätigkeit verstanden, egal welchen zeitlichen Umfang sie hat. Sind Sie ...

1. voll erwerbstätig
2. teilzeitbeschäftigt
3. in Altersteilzeit
4. geringfügig erwerbstätig in einem Mini Job
5. in einem "Ein-Euro-Job" (bei Bezug von Bürgergeld)
6. gelegentlich oder unregelmäßig beschäftigt
7. in einer beruflichen Ausbildung/Lehre
8. in Umschulung
9. im freiwilligen Wehrdienst/Bundesfreiwilligendienst/ Freiwilliges Soziales Jahr o.ä.
10. nicht erwerbstätig
11. Keine Angabe

Filter: lt. Frage 24 Pos. 2, 4, 6, 8 und 10

Frage 25

Zu welcher der folgenden Gruppen gehören Sie?

1. Schüler(in) an einer allgemeinbildenden Schule
2. Student(in);
3. Rentner(in), Pensionär(in) im Vorruhestand
4. Arbeitslos
5. in Mutterschafts-, Erziehungsurlaub, Elternzeit oder sonstiger Beurlaubung
6. Hausfrau, Hausmann
7. aus anderen Gründen nicht erwerbstätig
8. nichts davon trifft zu
9. Keine Angabe

Frage 26

Welchen höchsten Schul- bzw. Hochschulabschluss haben Sie? Bzw. an Schüler: Welchen allgemeinbildenden Schulabschluss streben Sie an?

1. ohne Haupt-/Volksschulabschluss
2. Haupt-/Volksschulabschluss
3. Mittlere Reife, Realschulabschluss, Fachschulreife

4. Abschluss der Polytechnischen Oberschule (8./10. Klasse)
5. Fachhochschulreife, Abschluss einer Fachoberschule
6. Abitur, allgemeine oder fachgebundene Hochschulreife
7. Fach-/Hochschulstudium
8. anderer Schulabschluss
9. Keine Angabe

Frage 27

Wie viele Personen leben ständig in Ihrem Haushalt, Sie selbst eingeschlossen? Denken Sie dabei bitte auch an alle im Haushalt lebenden Kinder.

1. [NUMERISCHEEINGABE]
2. Keine Angabe

Frage 28

Und wie viele Kinder unter 14 Jahren leben in Ihrem Haushalt?

1. [NUMERISCHEEINGABE]
2. Keine Angabe

Filter: Frage 28 >1

Frage 29

Und sind Sie in Ihrem Haushalt diejenige Person, die am meisten zum Haushaltseinkommen beiträgt?

1. Ja
2. Nein
3. Keine Angabe

Frage 30

Wie hoch ist das monatliche Nettoeinkommen Ihres Haushaltes insgesamt? Wir meinen damit die Summe, die sich ergibt aus Lohn, Gehalt, Einkommen aus selbständiger Tätigkeit, Rente oder Pension, jeweils nach Abzug der Steuern und Sozialversicherungsbeiträge. Rechnen Sie bitte auch die Einkünfte aus öffentlichen Beihilfen, Einkommen aus Vermietung, Verpachtung, Wohngeld, Kindergeld und sonstige Einkünfte hinzu.

1. Keine Angabe
2. unter 500 EUR
3. 500 bis unter 1.000 EUR
4. 1.000 bis unter 1.500 EUR
5. 1.500 bis unter 2.000 EUR
6. 2.000 bis unter 2.500 EUR
7. 2.500 bis unter 3.000 EUR
8. 3.000 bis unter 3.500 EUR

9. 3.500 bis unter 4.000 EUR
10. 4.000 bis unter 4.500 EUR
11. 4.500 Euro und mehr

Filter: monatliche Nettoeinkommen Ihres Haushaltes (Frage 30=11)

Frage 31

Und nennen Sie bitte auch, ob das monatliche Haushalts-Nettoeinkommen in einer der folgenden Kategorien liegt.

1. Keine Angabe
2. 4.500 bis unter 5.000
3. 5.000 bis unter 5.500
4. 5.500 bis unter 6.000
5. 6.000 bis unter 6.500
6. 6.500 bis unter 7.000
7. 7.000 bis unter 7.500

7.500 und mehr

Frage 32

Wie sind Sie krankenversichert?

1. Gesetzlich
2. Privat
3. Privat mit Beihilfe
4. Sonstiges
5. nicht versichert
6. keine Angabe

Frage 33

An dieser Stelle wurde im Rahmen der Studie die deutsche Übersetzung des EQ-5D-5L der EuroQol Research Foundation eingesetzt, um den aktuellen Gesundheitszustand der Teilnehmenden zu erfassen. Der vollständige Wortlaut der einzelnen Dimensionen sowie der Antwortoptionen darf aufgrund urheberrechtlicher Vorgaben nicht im Rahmen dieser Veröffentlichung wiedergegeben werden. Für die genaue Formulierung der Items wird auf das offizielle, lizenzierte Instrument der EuroQol Research Foundation verwiesen.

Frage 34

Zur zusätzlichen Einschätzung des aktuellen Gesundheitszustands wurde im Rahmen der Studie die deutsche Version der EQ-5D-5L Visuellen Analogskala (EQ-VAS) verwendet. Die Reproduktion der Skala und ihrer Instruktionen ist aus urheberrechtlichen Gründen nicht zulässig. Eine Beschreibung der EQ-VAS findet sich im offiziellen Material der EuroQol Research Foundation.

Copyright-Hinweis:© EuroQol Research Foundation. EQ-5D™ is a trade mark of the EuroQol Research Foundation. Germany (German) v1.0

Frage 35

Zum Abschluss der Befragung haben Sie die Möglichkeit, uns Rückmeldungen zu geben. Wenn Sie noch irgendwelche ergänzenden Anmerkungen haben, freuen wir uns sehr über Ihre Rückmeldung in diesem Feld.
[Eingabefeld für offene Frage]

Vielen Dank für Ihre Zeit!
