# Supplementary material for: Does Digital Make a Difference? Willingness‐to‐Pay for Digital Versus Offline Weight Loss in Germany
Source: Obes Sci Pract. 2026 May 21;12(3):e70151. doi: 10.1002/osp4.70151 (PMC13240293; doi:10.1002/osp4.70151)
Supplement: Supplementary file 2 — Supporting Information S2 [file OSP4-12-e70151-s001.pdf]

Supporting information 2: Operationalization of the descriptive values

Summary: This file provides an overview of the operationalization and coding of selected descriptive variables used in the empirical analyses, including constructed sociodemographic indicators, income measures, digital affinity indices, health status (EQ-5D-5L), and contextual factors related to medical care and digital infrastructure.

**Variable**	**Operationalization**
Education	• No school-leaving qualification (less than 9 years)<br>ohne Haupt-/Volksschulabschluss (unter 9 Jahren)<br>• Lower secondary school-leaving qualification (9 years)<br>Haupt-/Volksschulabschluss (9 Jahre)<br>• Intermediate secondary school-leaving qualification (10 years)<br>Mittlere Reife, Realschulabschluss, Fachschulreife (10 Jahre)<br>• Polytechnic secondary school-leaving qualification (10 years)<br>Abschluss der Polytechnischen Oberschule (10 Jahre)<br>• Upper secondary school-leaving qualification (12 years)<br>Fachhochschulreife, Abschluss einer Fachoberschule (12 Jahre)<br>• General or subject-specific higher education entrance qualification (13 years)<br>Abitur, allgemeine oder fachgebundene Hochschulreife (13 Jahre)<br>• Tertiary degree / Higher education degree (16 years)<br>Fach-/Hochschulstudium (16 Jahre)
Income	Net household income is divided by the total weight of the household, which is calculated using weights of 1.0 for the first adult, 0.5 for other adults and young people aged 14 and over and 0.3 for children under 14.<br>Income was categorized in binary form using the median net equivalent household income of 2400€.
Digital Affinity	The index for digital affinity is calculated by the weighted sum of the scores from four questions.<br>1. use of apps in general (weighting: 0.1): Yes (+1), No (-1), No answer (0).<br>2. use of health apps (weighting: 0.2): Yes (+1), No (-1), No answer (0).<br>3. attitude towards new apps (weighting: 0.3): Scale from very positive (+1) to very negative (-1).<br>4. knowledge of using apps (weighting: 0.4): Scale from very good (+1) to very poor (-1).
Health (EQ-5D-5L)	Calculated according to the Value Set Germany
Medical care structure	Very poor (1)<br>Poor (2)<br>Neutral (3)<br>Good (4)<br>Very good (5)
Digital infrastructure	Very poor (1)<br>Poor (2)<br>Neutral (3)<br>Good (4)<br>Very good (5)
